# Correlations between Sleep Bruxism and Temporomandibular Disorders

**DOI:** 10.3390/jcm9020611

**Published:** 2020-02-24

**Authors:** Brigitte Ohlmann, Moritz Waldecker, Michael Leckel, Wolfgang Bömicke, Rouven Behnisch, Peter Rammelsberg, Marc Schmitter

**Affiliations:** 1 Department of Prosthodontics, University of Heidelberg, Im Neuenheimer Feld 400, 69120 Heidelberg, Germany; moritz.waldecker@med.uni-heidelberg.de (M.W.); michael.leckel@med.uni-heidelberg.de (M.L.); Wolfgang.Boemicke@med.uni-heidelberg.de (W.B.); peter.rammelsberg@med.uni-heidelberg.de (P.R.); 2 Institute of Medical Biometry and Informatics (IMBI), University of Heidelberg, Im Neuenheimer Feld 130.3, 69120 Heidelberg, Germany; behnisch@imbi.uni-heidelberg.de; 3 Department of Prosthodontics, University of Würzburg, Pleicherwall 2, 97070 Würzburg, Germany; schmitter_M@ukw.de

**Keywords:** sleep bruxism, TMD, electromyographic/electrocardiographic data

## Abstract

The aim of this study was to identify correlations between sleep bruxism (SB) and temporomandibular disorders (TMD) as diagnosed by means of the research diagnostic criteria for temporomandibular disorders (RDC/TMD). Sleep bruxism was diagnosed on the basis of I) validated questionnaires, II) clinical symptoms, and III) electromyographic/electrocardiographic data. A total of 110 subjects were included in the study. Fifty-eight patients were identified as bruxers and 52 as nonbruxers. A psychosocial assessment was also performed. An RDC/TMD group-I diagnosis (myofascial pain) was made for 10 out of 58 bruxers, whereas none of the nonbruxers received a diagnosis of this type. No significant differences were found between bruxers and nonbruxers with regard to RDC/TMD group-II (disc displacement) and group-III (arthralgia, arthritis, arthrosis) diagnoses. Somatization was significantly more common among bruxers than nonbruxers. Multivariate logistic regression analysis revealed that somatization was the only factor significantly correlated with the diagnosis of myofascial pain. The results of this study indicate a correlation between myofascial pain, as diagnosed using the RDC/TMD, and somatization. It seems that somatization is a stronger predictor of an RDC/TMD diagnosis of myofascial pain than sleep bruxism is.

## 1. Introduction

Temporomandibular disorder (TMD) is the umbrella term for many clinical signs and symptoms of the structures of the masticatory system, including the masticatory muscles and the temporomandibular joints (TMJs). It has been suggested that many factors contribute to the development of TMD, and it is commonly assumed that bruxism is one of the major risk factors for temporomandibular disorders [1]. Because both conditions are prevalent (bruxism: 8–31% [2]; TMD: 10% [3]) and both are associated with subsequent medical costs, the correlation between bruxism and TMD is of great interest to researchers and clinicians.

Many clinical studies have investigated this correlation in recent years, producing a range of contradictory results and conclusions. For example, whereas some studies have established that bruxism is a significant risk factor for TMD pain [4,5] or clinically diagnosed disc displacement [6], others have concluded that no association exists between bruxism and functional [7,8] or muscle [9] symptoms. This contentious relationship between bruxism and TMD is due to the wide range of symptoms, diagnoses, and examination methods associated with both disorders. According to the International Classification of Sleep Disorders (ICDS-3), the criteria for the classification of sleep bruxism (SB) include the presence of regular or frequent tooth-grinding sounds during sleep and the presence of one or more of the following clinical signs: abnormal tooth wear consistent with reports of tooth grinding during sleep, and/or transient morning jaw-muscle pain or fatigue, and/or temporal headache, and/or jaw-locking upon awakening consistent with reports of tooth grinding during sleep [10]. However, most studies have been based on self-reported bruxism [11,12,13,14], but it is important to note that the validity of self-reported bruxism is controversial. This is because self-reporting alone has been blamed for an overestimation of the number of patients with bruxism. Moreover, patients themselves believe that bruxism is associated with TMD [15], possibly based on information received from their dentist. One study found that self-reporting of bruxism was substantially higher among individuals with painful TMD than among controls, even though the prevalence of SB as diagnosed by polysomnography (PSG) did not differ between the two groups [5]. Interestingly, almost 40% of those with TMD pain had been told by their dentist that they had SB.

It has been argued that a combination of instrument-based and noninstrument-based methods might be the best option for the diagnosis of SB [16]. Consequently, bruxism studies that are based on data from diagnostic devices in addition to other diagnostic methods might be expected to yield consistent findings. Yet, studies using PSG or electromyography (EMG) data have also produced inconsistent results, ranging from a positive association between bruxism and TMD pain [7], no association [5], and even a negative association [17,18]. Regarding function-related TMD diagnoses, instrument-based SB studies are rare, and the few that have been conducted have produced contradictory results [7,19].

The aim of this study was, therefore, to explore possible correlations between sleep bruxism (SB; diagnosed on the basis of questionnaire results, clinical signs, and EMC/PSG data (Bruxoff^®^ device, Allder, Moncalieri (To), Italy) and temporomandibular disorders as diagnosed using the research diagnostic criteria for temporomandibular disorders (RDC/TMD). A psychosocial assessment was also carried out that included the graded chronic pain scale, the jaw disability index, a depression scale, and the assessment of somatization.

## 2. Experimental Section

### 2.1. Study Design

This study was approved by the local Ethics Committee, protocol no. S-312/2014. All subjects were given information about the procedure and the possible risks and benefits of the study. All subjects gave informed consent.

### 2.2. Participants

One hundred and ten (110) study participants were preliminary selected from a clinical study evaluating dental restorations among patients with and without sleep bruxism (clinical trial no. NCT03039985). Thus, all participants included in the study were in need of a posterior crown with a natural antagonist. Participants under the age of 18 or who were not permitted to take out a contract were excluded from the study, as were patients who were pregnant or lactating, had acute neuropsychiatric diseases, hemorrhagic diathesis, or a heart pacemaker, or who had a known allergic reaction to the materials used.

### 2.3. Diagnosis of Sleep Bruxism

The presence of sleep bruxism was diagnosed in accordance with the criteria of the American Academy of Sleep Medicine [20] and was based on I) self-reporting of bruxism, II) a clinical examination to evaluate bruxism signs, and III) data from a portable EMG/ECG recorder (Bruxoff^®^) as a replacement for PSG.

I) Two questionnaires were used for the self-reporting of bruxism:

(i) a questionnaire by Paesani et al. [21] and (ii) a structured interview by Raphael et al. [5]. In this study, self-reported bruxism was recorded as “yes” or “no” if both questionnaires yielded identical results [22].

II) Participants were examined for the presence of four clinical signs of bruxism:

(i) abnormal tooth wear, (ii) impressions of teeth in the buccal region, (iii) impressions of teeth on the tongue, and (iv) hypertrophy of the masseter muscle. In this study, clinical signs of bruxism were deemed present if one of the four items was answered “yes.”

III) A portable bruxism device (Bruxoff^®^) was used to record data over five nights.

A diagnosis of bruxism was confirmed if more than two SB episodes were determined per hour of sleep during at least one night’s sleep. This was defined as the cut-off for moderate sleep bruxism (i.e., >2 SB episodes per hour of sleep), and the cut-off for severe sleep bruxism was set at more than four SB episodes per hour of sleep.

Patients were included in the bruxism study group if all three items (I–III) were answered “yes.” If all three items were answered “no”, they were included in the nonbruxism study group.

Patients who had any discrepancies were excluded.

### 2.4. Diagnosis of Temporomandibular Disorders (RDC/TMD)

All subjects underwent a clinical examination based on the Axis-I protocol of the RDC/TMD (including the appropriate diagnostic aspects) [23], resulting in the following diagnoses:

**Group I: muscle disorders**

*Ia. Myofascial pain without limited opening:*

- Self-reporting of pain in the jaw, face, preauricular area, or inside the ear;
- Pain reported in response to palpation of ≥3 of the following muscles sites: posterior/middle/anterior temporalis, origin/body/insertion of the masseter, posterior mandibular region, submandibular region, lateral pterygoid area, and tendon of the temporalis;
- At least one of the painful sites must be on the same side as the self-reported pain.

*Ib. Myofascial pain with limited opening:*

- Myofascial pain as defined in Ia;
- Pain-free unassisted mandibular opening <40 mm;
- Maximum assisted opening (passive stretch) ≥5 mm.

**Group II: disc displacements**

*IIa. Disc displacement with reduction:*

- Reproducible clicking in TMJ on opening and closing with ≥5 mm interincisal distance and which is eliminated on protrusive opening; or
- Reproducible clicking in TMJ on opening or closing, and click during lateral excursion or protrusion.

*IIb. Disc displacement without reduction with limited opening and*

*IIc. Disc displacement without reduction, without limited opening:*

- Both diagnoses were made by use of the RDC/TMD diagnostic algorithm [23] and inlcuded the following parameters: history of significant limitation in opening, maximum unassisted opening, passive stretch, and contralateral and uncorrected deviation.

**Group III: arthralgia, osteoarthritis, and osteoarthrosis**

*IIIa. Arthralgia:*

- Pain in one or both joint sites during palpation; and
- Self-reporting of one or more of the following types: pain in the region of the joint, pain in the joint during maximum unassisted opening, pain in the joint during assisted opening, and pain in the joint during lateral excursion;
- Coarse crepitus must be absent.

*Group IIIb. Osteoarthritis:*

- Arthralgia as defined in IIIa;
- Coarse crepitus in the joint.

*Group IIIc. Osteoarthrosis:*

- Neither self-reported pain nor pain during palpation;
- Coarse crepitus in the joint.

In addition, all subjects underwent a psychosocial assessment based on Axis II of the RDC/TMD, including the graded chronic pain scale [24], the jaw disability index [23], a depression scale [25], and the assessment of somatization [26].

### 2.5. Statistical Analysis

Data were evaluated by use of statistical software (SPSS 25; IBM Corp., New York, United States and R Project for statistical computing, version 3.6.1 (2019-07-05)). Chi-squared tests were used to evaluate categorical data, and multivariate logistic regression analysis was used to identify risk factors for the RDC/TMD Axis I diagnoses. Statistical significance was set at *α* = 0.05.

## 3. Results

During the study recruitment period, 110 subjects were enrolled, comprising 45 males (mean age: 54 years; SD: 11.9) and 65 females (mean age: 50 years; SD: 12.8). Fifty-eight patients were identified as bruxers (SB episodes >2) and 52 subjects as nonbruxers. The 58 bruxers were further subclassified as 23 moderate bruxers (SB episodes >2 and ≤4) and 35 severe bruxers (SB episodes >4).

Table 1 shows the raw data regarding the correlation between sleep bruxism and the RDC/TMD Axis-I diagnosis. Significant differences were detected between bruxers and nonbruxers regarding the group-I diagnosis of myofascial pain (*p* = 0.011). None of the subjects in the nonbruxer group had myofascial pain with or without limited mouth opening, whereas a total of 10 subjects (including nine females) in the bruxer group (17.2%) had myofascial pain with or without limited mouth opening.

Regarding the joint-related RDC/TMD group-II diagnosis, eight subjects in the bruxer group (13.8% including seven females) and six subjects in the nonbruxer group (11.5%, including five females) received this type of diagnosis, with no significant differences observed between the bruxers and nonbruxers (*p* = 0.930). Four patients in the bruxer group received a group-II diagnosis for both TMJs, whereas all patients in the nonbruxer group only received a diagnosis for one of the two TMJs.

A group-III diagnosis was made for only four subjects in the bruxer group (6.9%, including three females) and three female subjects in the nonbruxer group (5.7%), including two subjects who received a diagnosis of osteoarthrosis in both TMJs. No significant differences were found between the bruxers and nonbruxers (*p* = 0.789).

The correlations between sleep bruxism and depression, somatization, and pain (as assessed using the graded chronic pain scale) are presented in Table 2. The results indicate a correlation between somatization and sleep bruxism (*p* = 0.0083).

Table 3 shows the correlation between the results of the jaw disability index and sleep bruxism. In the nonbruxer group, 98% of subjects had no limitations as measured by the jaw disability index. In the bruxer group, 69% of the moderate bruxer group and 60% of the severe bruxers reported no limitations.

The results of the multivariate logistic regression analysis e are given in Table 4, Table 5 and Table 6, showing a significant correlation between somatization and myofascial pain (*p* = 0.049) and between female gender and disc displacement (*p* = 0.030).

## 4. Discussion

The results of this study support the assumption that temporomandibular disorders are a complex condition. They also suggest that unspecific physical symptoms are a stronger predictor of an RDC/TMD diagnosis of myofascial pain than a diagnosis of sleep bruxism is.

The most common reason to seek medical advice is the symptom of pain. Two main theories exist regarding the development of pain: the vicious pain theory and the pain adaption model [27]. The vicious pain theory assumes that sleep bruxism causes pain that results in muscle spasms, thus leading to further pain. Starting from this point, one would expect SB to be more severe among symptomatic participants than among controls. This is consistent with most of the studies in which bruxism was diagnosed on the basis of self-reporting, indicating a strong association of TMD with bruxism [28].

In contrast, if a device is used to diagnose bruxism, then this clear association does not exist. Instrument-based studies have shown that bruxism variables do not differ between symptomatic and nonsymptomatic subjects [29,30], and that most participants do not even exhibit SB [5]. In addition, it seems that SB patients without TMD have more SB episodes and a longer duration of bruxism per hour of sleep [29], and that experimentally induced TMD pain appears to reduce SB activity among healthy individuals [31]. All these results are more consistent with the second major pain theory, the pain adaption model [27], which suggests that muscle pain leads to a reduction in muscle activity, thus protecting the muscle from further injury.

In our study, however, myofascial pain was only detected among subjects with sleep bruxism. This is more consistent with the vicious pain theory and the observations of Rosetti et al. [32] and Santiago et al. [33], but contradicts the results of other instrument-based studies [5,29]. Nonetheless, this positive association of myofascial pain with SB disappeared as soon as the effect of somatization was included as a possible risk factor for myofascial pain in statistical analysis. This is in accordance with the results of studies investigating the association of TMD pain with psychosocial status. Subjects with painful TMD often exhibit an emotional personality with high to moderate levels of somatization and/or depression [9,29,34,35,36,37], suggesting that factors other than SB might contribute to TMD pain [38]. In agreement with the results of Muzalev et al. [37,39], the results of our study support the argument that myofascial pain cannot be explained by a simple linear cause-and-effect relationship between pain and sleep bruxism. Our results are also consistent with PSG studies showing that sleep bruxism is not positively associated with TMD among adults [40], as well as showing that somatization is a more important predictor for myofascial pain than sleep bruxism.

Nevertheless, some common limitations of SB and TMD studies must be kept in mind. First, the signs and symptoms of TMD fluctuate substantially [14], as do those of sleep bruxism [17]. This means that all instrument-based studies, including this one, can provide only a limited insight into these long-term, fluctuating conditions. To understand TMD development and the relevant risk factors (in particular, if the effect of SB on TMD development is time-dependent [41]), longitudinal studies involving TMD diagnosis criteria and patients with verified bruxism are required. Because each clinical bruxism symptom seems to relate to different aspects of jaw motor activity [42], this should also be investigated in long-term clinical studies. Furthermore, the available data suggest that some individuals are more vulnerable to TMD and TMD pain than others, possibly as a result of genetic risk factors [43]. This might explain the contradictory results of studies so far and should be taken into account when selecting study subjects.

Regarding a TMJ-related RDC/TMD diagnosis, few data are available. Unlike the studies by Blanco Aguilera et al. [8] and Rosetti et al. [7], this study could not prove a clear correlation between SB and a TMJ-related RDC/TMD diagnosis. In contrast to the results of Baba et al. [19], however, a strong correlation was observed between the duration of masseter EMG and TMJ clicking. These differences might be due to the different TMD diagnosis schemes used. Baba et al. used a nonvalidated TMD diagnostic method, and any disagreements between the subject and examiner with regard to the diagnosis of TMJ clicking were recorded as “no”. This might have affected the results.

In addition to a diagnosis of bruxism, it is generally accepted that gender is associated with temporomandibular disorders [44]. Although the multiple regression model with myofascial pain as the dependent variable did not reveal statistically significant differences between males and females, the odds ratio of 11.39 indicates that this effect is not negligible. The significant differences concerning disc displacements also indicate an effect of female gender.

Our study also has its weaknesses. First of all, the available data suggest that the number of bruxism events is less important than the general amount of EMG activity, which seems to be higher among individuals with TMD than among controls [45]. This suggests that the duration and amount of muscle activity are better predictors of a SB diagnosis than the number of events, and should therefore be used instead of the diagnosis cut-off points used in this study [46]. In addition, subjects were selected based on the criterion “in need of a molar crown”, irrespective of their TMD and bruxism status or a TMD-relevant medical history, for example, Sjögren-syndrome [47]. None of the TMD-subjects in this study had relevant diseases in their medical history; nevertheless, it should be kept in mind that the number of participants with an RDC/TMD diagnosis was small, and the results should therefore be interpreted with appropriate caution. Furthermore, this study only investigated sleep bruxism, and the effect of awake bruxism was not tested. A study by Reissmann et al. [48] showed that sleep and awake bruxism are not separate conditions but interact additively, and this should be taken into consideration in further research. The main weakness of the current study is possibly the use of the Bruxoff^®^ device. Although this portable bruxism device demonstrated high sensitivity in detecting sleep bruxism [49], it cannot be considered a reliable replacement for the gold standard of PSG, because it does not feature audio-video recording or electroencephalogram monitoring [50]. Thus, it cannot register visual or auditory activity, and it remains unclear whether there are periods of WASO (wake after sleep onset) during recording. Finally, although the specificity and sensitivity of an RDC/TMD or DC/TMD diagnosis for myofascial pain are acceptable, it must be kept in mind that a RDC/TMD TMJ-related diagnosis is not definitive without appropriate gold-standard use of magnetic resonance imaging of the TMJ [51].

## 5. Conclusions

The results of this study support the assumption that myofascial pain cannot be explained solely by a simple cause-and-effect relationship between sleep bruxism and myofascial pain. Nonspecific physical symptoms seem to be a stronger predictor than sleep bruxism for the RDC/TMD diagnosis of myofascial pain. Longitudinal studies are needed to investigate these results further, ideally involving patients with verified diagnoses of bruxism and TMD.

## Figures and Tables

**Table 1:** Correlation between moderate (>2 and ≤4 SB episodes/h) and severe (>4 SB episodes/h) sleep bruxism and a diagnosis in accordance with the RDC/TMD.

RDC/TMD Diagnosis	Moderate Bruxer (*n* = 23)	Severe Bruxer (*n* = 35)	Nonbruxer (*n* = 52)	*p*-Value
**Group I**				0.011
No group-I diagnosis	18	30	52	
I a	4	5	0	
I b	1	0	0	
**Group II, dichotomous**				0.930
Yes	3	5	6	
No	20	30	46	
**Group II, detailed**				NA
No group-II diagnosis, right TMJ	21	31	48	
II a, right TMJ	2	4	4	
II b, right TMJ	0	0	0	
II c, right TMJ	0	0	0	
No group-II diagnosis, left TMJ	21	31	50	
II a, left TMJ	2	4	2	
II b, left TMJ	0	0	0	
II c, left TMJ	0	0	0	
**Group III, dichotomous**				0.789
Yes	1	3	3	
No	22	32	49	
**Group III, detailed**				NA
No group-III diagnosis	22	32	50	
III a	1	2	0	
III b	0	0	0	
III c	0	1	2	

Group I: Myofascial pain without (I a) or with (I b) limited mouth opening. Group II: TMJ disc displacements with reduction (II a), without reduction with limited mouth opening (II b), and without reduction without limited mouth opening (II c). Group III: TMJ arthralgia (III a), osteoarthritis (III b), or osteoarthrosis (III c). RDC/TMD = research diagnostic criteria for temporomandibular disorders. SB = sleep bruxism. TMJ = temporomandibular joint. NA = not available. Significant *p*-values are marked in bold.

**Table 2:** Correlation between moderate (>2 and ≤4 SB episodes/h) and severe (>4 SB episodes/h) sleep bruxism and depression, somatization, and pain (GCP).

	Moderate Bruxer (*n* = 23)	Severe Bruxer (*n* = 35)	Nonbruxer (*n* = 52)	*p*-Value
**Depression**				0.1734
Normal	20	28	48	
Abnormal	2	4	0	
Not evaluable	1	3	3	
**Somatization**				0.0083
Normal	13	23	46	
Possibly Abnormal	4	5	4	
Abnormal	6	7	1	
**Pain (GCP)**				NA
0	15	23	50	
I	7	7	2	
II	1	3	0	
III	0	1	0	
IV	0	0	0	

GCP = graded chronic pain scale. NA = not available. Significant *p*-values are marked in bold.

**Table 3:** Self-reported limitations caused by problems with the TMJ or muscles (12 items from the jaw disability index).

Score of Limitations	Moderate Bruxer (*n* = 23)	Severe Bruxer (*n* = 35)	Nonbruxer (*n* = 52)	*p*-Value
				NA
0 = no limitations	16	21	51	
1	4	6	1	
2	2	2	0	
3	0	2	0	
4	1	2	0	
5	0	0	0	
6	0	0	0	
7	0	0	0	
8	0	0	0	
9	0	1	0	
10	0	0	0	
11	0	1	0	
12	0	0	0	

TMJ = temporomandibular joint. NA = not available.

**Table 4:** Multivariate logistic regression model with RDC/TMD group I (myofascial pain) as the dependent variable. Estimates of the model coefficients.

	Estimate	Standard Error	*z*-Value	Pr (>*z*)	OR
(Intercept)	−2.107	2.714	−0.777	0.437	0.122
Age	−0.042	0.049	−0.857	0.391	0.959
Gender, female	2.433	1.413	1.722	0.085	11.392
Somatization Possibly abnormal	1.150	1.126	1.021	0.307	3.157
Somatization Abnormal	2.889	1.467	1.969	0.049	17.970
Depression Abnormal	−0.512	1.463	−0.350	0.726	0.599
Depression Not evaluable	−16.585	6247.937	−0.003	0.998	0.000
Severe bruxer	−0.526	0.937	−0.562	0.574	0.591
Nonbruxer	−18.465	2292.787	−0.008	0.994	0.000

OR = odds ratio. Significant *p*-values are marked in bold.

**Table 5:** Multivariate logistic regression model with RDC/TMD group II (disc displacements) as the dependent variable. Estimates of the model coefficients.

	Estimate	Standard Error	*z*-Value	Pr (>*z*)	OR
(Intercept)	−4.468	1.915	−2.333	0.020	0.011
Age	0.036	0.031	1.164	0.244	1.037
Gender, female	1.819	0.838	2.169	0.030	6.163
Somatization Possibly abnormal	−1.279	1.145	−1.117	0.264	0.278
Somatization Abnormal	−0.375	1.224	−0.307	0.759	0.687
Depression Abnormal	−17.485	2647.809	−0.007	0.995	0.000
Depression Not evaluable	−15.833	2631.774	−0.006	0.995	0.000
Severe bruxer	0.343	0.893	0.384	0.701	1.409
Nonbruxer	−0.934	0.887	−1.053	0.292	0.393

OR = odds ratio. Significant *p*-values are marked in bold.

**Table 6:** Multivariate logistic regression model with RDC/TMD group III (arthralgia, osteoarthritis and osteoarthrosis as the dependent variable. Estimates of the model coefficients.

	Estimate	Standard Error	*z*-Value	Pr (>*z*)	OR
(Intercept)	−4.201	2.536	−1.657	0.098	0.015
Age	−0.013	0.040	−0.324	0.746	0.987
Gender, female	1.421	1.219	1.165	0.244	4.140
Somatization Possibly abnormal	0.549	1.240	0.443	0.658	1.732
Somatization Abnormal	1.854	1.367	1.356	0.175	6.385
Depression Abnormal	−0.584	1.630	−0.358	0.720	0.558
Depression Not evaluable	−15.110	2524.621	−0.006	0.995	0.000
Severe bruxer	1.002	1.302	0.770	0.442	2.723
Nonbruxer	0.936	1.399	0.669	0.504	2.550

OR = odds ratio. Significant *p*-values are marked in bold.
